# Engaging with Community Advisory Boards (CABs) in Lusaka Zambia: perspectives from the research team and CAB members

**DOI:** 10.1186/s12910-015-0031-y

**Published:** 2015-06-03

**Authors:** Alwyn Mwinga, Keymanthri Moodley

**Affiliations:** Centre for Medical Ethics and Law, Stellenbosch University, Stellenbosch, South Africa

**Keywords:** Community engagement, Community advisory board, Community based participatory research, research team

## Abstract

**Background:**

The use of a Community Advisory Board (CAB) is one method of ensuring community engagement in community based research. To identify the process used to constitute CABs in Zambia, this paper draws on the perspectives of both research team members and CAB members from research groups who used CABs in Lusaka. Enabling and restricting factors impacting on the functioning of the CAB were identified.

**Methods:**

All studies approved by the University of Zambia Bioethics Research Committee (UBNZABREC) from 2008 – 2012 were reviewed to identify those studies that were likely to include a CAB. Eight teams with studies that included a CAB were identified. For each of these studies, consent was obtained to conduct an informal interview with a research team member and to obtain contact details for one CAB member. In total 14 interviews were conducted with 8 research team members and 6 CAB members from 12–30 August 2013.

**Results:**

Identification of potential CAB members from the community and their participation in developing the terms of reference for CABs was perceived to have contributed to the success of the CAB. Due to the trust that the community had in members of their community the CABs were then in a stronger position to influence community participation in the research. Training of CAB members was identified as a factor that enhanced the functioning of a CAB. Lack of commitment and low literacy levels of CAB members posed a threat to the role of the CAB. Although compensation in the form of a stipend was not provided, CAB members were provided with transport reimbursements for attending meetings.

**Conclusions:**

Selection of CAB members from within the community contributed to community confidence in the CAB, enhancing its ability to act as an effective link between study team and community. This contributed positively to the conduct of the study and enhanced community awareness and acceptance of the research. However, establishment of study specific CABs has the potential to compromise CAB independence due to support provided by the research team in the form of transport reimbursements and other forms of support. Consideration should be given to establishing community wide Community Advisory Boards that could function across a range of studies to increase independent objective decision-making.

## Background

The concept of community based research provides an approach that ensures the inclusion of the community as true partners in all phases of the research enterprise based on key principles that include, amongst others, the recognition of the community as a unit, the use of community structures and strengths, and sharing access to information and knowledge with all partners [1]. In the context of HIV/AIDS research the U.S. National Institutes of Health (NIH) first mandated the use of Community Advisory Boards (CABs) in clinical trials in 1987 [2] in response to AIDS activism in the 1980s. The involvement of CABs is now a requirement for all NIAID sponsored programs [3] and the mechanisms for forming CABS are an integral component of the ethical review process.

The roles that have been documented in the literature for the Community Advisory Board include functioning as a liaison between the researchers and the community, providing information to the community about the study including their rights to consent, generally improving the informed consent process, ensuring human subjects protection, advocacy for fair compensation for trial related injuries, protection of minorities and involvement in disseminating results to communities [2, 4]. Other functions that could be performed by CABs include protocol development and review, identification and referral of potential study participants, and identification of methods to trace lost participants [2, 4–6]. In addition CABs have served to identify community priorities, needs and interests; set research priorities; provide input or resources for research activities; identify community members to serve on project steering committees and promote community support for and involvement with research [7, 8]. Building capacity in the community and developing a culture of human rights were additional roles [5]. The use of CABs has been associated with a sense of mutual trust and collective ownership when used in studies with long term follow-up.

The processes by which community consultations have occurred and CAB members are identified have varied across different sites and country contexts but have primarily focused on obtaining representation from organizations within the community [3, 5, 6, 8]. Community consultations to determine influential individuals and groups in the community can aid both the transparency of selection [9] and the acceptability of selected community representatives [10].

Different modes of selecting CAB members are evident. One review of Community Advisory Boards in 6 research sites identified two models for selection of CAB membership which are a “broad community model” and a “population specific “model [11]. The broad community model, found in Thailand and Zimbabwe, included a cross section of individuals from the broader community and included representatives based on the particular needs of the community. In the Thai model local government and police were included in the CAB due to the existing social climate when the CAB was established [6]. The population specific model consisted of representation from a limited section of the population and reflected the needs of the particular group at risk that were the subject of the protocol. This model was found in Peru, Los Angeles and Philadelphia [11]. A study of CABs in South Africa [5] revealed the existence of three selection process models; namely purposeful selection whereby members of the CAB were chosen from organizations with an interest in the research; election through a democratic process; and a mixed model that included elements of both the purposeful selection and election models.

It is fundamentally important that the CAB is able to carry out its functions independently of the research team in order to protect the community from any unethical research practices. A factor that has the potential to negatively influence this independence is the mechanism for compensating CAB members for their time and providing resources for the functioning of the CAB [5, 7]. Even though the provision of stipends or honoraria by the research institute [2, 8] can serve as a form of recognition for the contribution of the CAB member, in resource limited communities even providing relatively small amounts of money in the form of transport reimbursements does have the potential for undue influence [7]. Identification of CAB members who are already involved in other community based organizations or otherwise fully employed is one strategy that may be used to avoid the need for incentives. However, although this may improve sustainability of the CAB [12], including full-time salaried staff as members of the CAB may compromise the time they have to commit to CAB functions.

Zambia has been a recipient of international collaborative funding since the mid-1980s and several of these studies have involved the establishment of CABs, in line with both the requirement of the Research Ethics Committee as well as the funding organizations such as the NIH. This paper describes the processes that were used to set up CABs in Lusaka, Zambia, the methods used to determine the membership and select members, the functions carried out by the CAB, and the process used to develop Terms of Reference. The paper explores factors that positively contributed to the functioning of the CAB (enabling factors) or that had a negative impact on the CAB functions (restricting factors) from the perspective of the research team members and CAB members.

## Methods

The study was approved by the University of Zambia Biomedical Research Ethics Committee (Ref No 014-05-13) and the Health Research Ethics Committee 2 of the Stellenbosch University (Ref S13/04/079). The research was carried out in accordance with the Helsinki Declaration.

A purposive sampling method was used to identify and select studies with established CABs. For each study a separate interview was carried out with a member of the research team and the CAB respectively in order to obtain both perspectives.

### Identification of studies with a CAB

All studies approved by the University of Zambia Bioethics Research Committee between 2008 and 2012 were reviewed. A file containing third copies of all approval letters was reviewed and potential studies selected based on key words and phrases in the title such as “double blind”, “placebo controlled”, “trial”, “safety and efficacy”, “assessment of the use of”, “intervention trial” as possible indicators of a clinical trial. The ethics committee reference number of the identified studies was used to extract the study file from which the name and contact details of the Principal Investigator (PI) and other investigators. Telephonic contact was made with the Principal Investigator or other contact person to confirm whether the study included a CAB. For studies meeting the criteria, permission was obtained from the PI or contact person to include the study as well as to obtain contact details of potential study team and CAB members to be interviewed.

### Interview process

An individual semi-structured interview was carried out with all who consented to take part in the study using an interview guide. The guide comprised a set of 12 questions for the research team member and 8 questions for the CAB member (Annex 1). Notes of the responses to each question, including verbatim responses to questions were taken during the interview. Data collection was completed once all available respondents had been interviewed.

### Data analysis

The responses to the questions were manually analysed using content analysis by the researcher. This was achieved by developing a table with columns for the responses to the following questions: details of study (Study/sponsor, details of study), details of respondent (role in study/CAB), inclusion of CAB in protocol, process followed to form the CAB, the process used to make the community aware of the study, determination of CAB membership , selection process for members of the CAB, development of Terms of Reference for the CAB, support provided to CAB, what worked well (enabling factors), what did not work well (restricting factors), and perceptions on the added value of the CAB to the study. A list of the responses to each question was developed, grouping similar responses together, and the frequency of responses for each question was noted. The responses are presented under the following broad areas; process for formation of CAB, support provided to the CAB, perspectives of the role of the CAB, Enabling and Restricting factors, and added value of the CAB.

## Results

### Identification of studies with Community Advisory Boards

Based on the review of the paper file containing third copies of all approval letters there were 942 studies approved by UNZABREC from 2008 – 2012 of which 27 studies were selected as potential clinical trials and 21 of these files were located. Following contact with the Principal Investigator or other named investigator it was determined that 11 of the studies had a CAB, eight of the studies did not have a CAB and two studies were withdrawn after REC approval. Details of an additional two studies with a CAB were provided to the investigator by one of the study teams. These studies had commenced prior to 2008.

### Selection of studies

The 11 studies identified from the search of the records of the research ethics committee were submitted by three distinct research groups. Four of the studies were conducted by one research group with each study having a study-specific CAB. Each CAB was represented on the group’s Central CAB within their Clinical Trials Unit (CTU). An interview was scheduled for each study specific CAB in the CTU. Two of the studies were submitted by another research group and one interview was scheduled with this group. Together with the one interview with the third research group and the two additional studies this provided a total of 8 possible Research team/CAB pairs. The studies included five drug or treatment trials, one observational study and two preparatory studies (Table 1). All studies were sponsored by international organizations.

**Table 1:** Characteristics of studies included in sample

Study Number	Type of Study	Sponsorship
1	Observational study on sexual behaviour in HIV positive clients	International Organization/Collaboration
2	Drug trial comparing safety and efficacy of two different regimens	International Organization/Collaboration
3	Drug treatment trial comparing outcomes of two treatment strategies	International Organization/Collaboration
4	Preparatory study, waiting ethical approval	International Organization/Collaboration
5	Community randomized trial of interventions to decrease prevalence of disease	International Organization/Collaboration
6	Treatment trial comparing two different treatment strategies	International Organization/Collaboration
7	Drug treatment trial to establish efficacy of drug	International Organization/Collaboration
8	Treatment trial and preparation for vaccine trial	International Organization/Collaboration

### Respondents

Interviews were conducted with a total of 14 out of the possible 16 respondents consisting of eight members of the research team and six members of the CAB. One CAB member was not contactable using the details provided while the other CAB member was unable to attend the scheduled meeting due to unforeseen circumstances and no other time was available for the interview. None of the people approached refused to participate in the study. The role of the research team members in the study were as follows; senior research nurses (two), Community Liaison (one), co-Study manager (one), community health educator (two), Co-PI (one) and Social Scientist (one). The roles of the CAB member were Chairperson (two), Secretary (one), and ordinary members (three) (Table 2).

**Table 2:** Characteristics of Respondents

Study No	Respondent type	Role in study
1	Study team	Senior Research Nurse
	CAB	CAB Member
2	Study team	Senior Research Nurse
	CAB	CAB Chairperson
3	Study team	Community Health Educator
	CAB	Secretary of CAB
4	Study team	Community Educator
	CAB	CAB Chairperson
5	Study team	Co-Study Manager
	CAB	CAB Member
6	Study team	Social Scientist
	CAB	CAB Member
7	Study team	Community Liaison
8	Study Team	Co-Investigator

### Interview process

Twelve of the interviews were conducted face to face and two by telephone due to the non-availability of one of the interviewees (research team member) and logistical difficulty to meet (CAB member). Signed informed consent was obtained for each face to face interview and verbal consent for the telephone interview following a discussion of the purpose and process for the study and providing time for the participant to read through the Patient Information Sheet for the face to face interview.

### Process for formation of CAB (Table 3)

**Table 3:** Characteristics of CAB and process for formation

Study No	CAB size	Was CAB included in Protocol?	Who decided on structure of CAB Membership?	How were CAB members selected?	Who developed ToR?
1	5	Yes	Research Team and Central CAB	Interviews of applicants responding to adverts	Central CAB
2	5	Yes	Research Team and Central CAB	Interviews of applicants responding to adverts	Central CAB
3	5	Yes	Research Team and Central CAB	Interviews of applicants responding to adverts	Central CAB
4	5	Yes	Research Team and Central CAB	Interviews of applicants responding to adverts	Central CAB
5	10 - 15	Yes, not fully developed	Research team	Study team and Neighbourhood health committee members	Study Team
6	5	No	Research and Clinic Staff	Neighbourhood health committee and other existing community groups	Study Team and CAB
7	42	Yes	Research Team with Community leaders and Stakeholders	Initially by Community Development Committee, later involved other stakeholders	Study team and community leaders
8		Yes	Research team and community members participating in study	Study team and community members participating in study	Study Team and Sponsor

The concept of a CAB was included in the protocol for seven of the eight studies while for the eighth study the idea of including a CAB came about as a result of discussions with the study team on strategies to improve community awareness, participation, and retention on the study. Table 3 below includes details for each study on who decided on the membership of the CAB, how the CAB members were selected and who developed the Terms of Reference for the CAB. The Research Team was involved in determining the structure of CAB membership in all the studies; this was done in conjunction with the Central CAB (4 studies), clinic staff (1 study), community leaders (1 study) or community members involved in the study (1 study). The selection of CAB members followed the “Broad community model” through either local advertisements and interviews of applicants (4 studies) or selection of members from existing community structures by research teams working with the members of these structures (3 studies) or clinic staff (1 study). Terms of Reference were developed by the Central CAB and adapted to the local situation for the 4 studies from the one research group. In the other studies, the terms of reference were developed either by the research team alone (one study), with the CAB and the research team (1 study), with community leaders (1 study) or with the sponsor (1 study). The size of the CAB varied from 5 members (5 studies) to 42 members (1 study).

### Support provided to the CAB

The research team provided training for the CAB members that included protocol specific training and training in research and ethics of research. Other support provided included transport reimbursement for attendance at meetings, refreshments for meetings, support for income generating activities for volunteer groups, capacity building for writing business proposals, support for community sensitization such as funds for drama groups and megaphones, T-shirts, gum boots and umbrellas as well as a computer for data collection (one study). No stipends were provided to the CAB members for their involvement.

*“The CAB members were provided with transport reimbursement only when they were invited to attend meetings at the research office, but not for meetings held within the clinic. As they were Neighbourhood Health Committee (NHC) members we did not want to introduce extra payments that they did not already get”. (Social Scientist)*

*“In addition to providing refreshments for meetings and transport refunds the CAB members were provided with training specific to the protocol as well as for principles of research in general”. (Co-PI)*

Providing opportunity for CAB members to attend international meetings was cited by five of the teams. Providing transport reimbursements was seen as both facilitating the work of the CAB or, possibly, leading to aligning the CAB more with the research team than the community.

### Perspectives on the role of the CAB (Table 4)

**Table 4:** Role of CAB

Function	Number of Responses	Number of Research Team responses	Number of CAB responses
Sensitize communities about the study	14	8	6
Function as a link between the community and the research team for information sharing and to improve the relationships	12	7	5
Review of proposal, questionnaire’s, draft study tools	10	6	4
Help to dispel rumours, clear misconceptions about the study, reduce stigma	8	4	4
Help in recruitment and mobilization of participants	3	2	1
Ensure protection of interests of the participants and that concerns are addressed	4	1	3
Help in improving retention of participants in the study	3	3	
Identify issues affecting participation in study, give advice on procedures	2	2	

The key function of the CAB from the perspective of all the respondents was noted to be that of sensitizing the communities about the research study. The CAB was also felt to be an important link between the research team and the community by 12 of the 14 respondents (7 research team, 5 CAB), helping to build relationships, and the development of trust by the community. This was attributed to the fact that the members of the CAB were part of the community and therefore known and trusted. The CAB was referred to as a “bridge” and a “belt” by two of the CAB members. The ability of the CAB to ensure acceptance by the community of the research was further demonstrated by the fact that 8 of the 14 respondents (4 research team and 4 CAB members) indicated an important role of the CAB in dispelling rumours and misconceptions of the study and also reducing stigma related to participation in the study.

*“The CAB was very useful to help dispel rumours and clear misconceptions as they are known by the community and the community trusts them”. (Senior Research Nurse)*

“*The CAB acts as the voice of the community and holds meeting with the community to address rumours, myths and misconceptions of the study”. (CAB chairperson).*

The ability of the CAB to influence the acceptance by the community of the research was related to the fact that the members of the CAB were part of the community, known and trusted.

*“It is important to choose members of the CAB from the community as they are known and trusted by the community and they can help to ensure acceptance of the research by the community”. (CAB member).*

Ten of the 14 respondents (6 research team, 4 CAB) recognized the key role of the CAB in reviewing the proposal, questionnaires and other study instruments.

*“The CAB was involved in the review of the study instruments and this helped to ensure that the questions were culturally acceptable to the community”. (Senior Research Nurse).*

*“The CAB reviews the protocols and informed consent documents before they are finalized and make sure that the concerns of the community are addressed”. (CAB Secretary).*

In addition to their role in dispelling rumours and misconceptions the CAB was noted to help mobilize communities and increase recruitment (3 responses) and improve retention in the study (3 responses).

*“Once the CAB began working our recruitment rate went up and we had better retention rates. Some of the study participants came to ask about the research after hearing about it from the CAB member in the community”. (Social Scientist)*

The CAB was also viewed as advocating for the interests of both the research team and the research participants.

*“The CAB should be pro-active in advocating for improved conditions for the participants such as increasing transport reimbursements. This would also help in retention in the study”. (Research Nurse).*

A positive role of the CAB in the study was seen to be their ability to identify issues affecting processes and procedures in the study and giving advice on how to improve these. Other functions included advocating for improved clinic conditions and additional services such as food supplements for patients.

*“The CAB was useful in providing advice to the research team on what to do and not to do in conducting the study”. (Social Scientist)*

*“One of the CAB members noticed that the physical arrangement in the clinic did not provide for confidentiality and after this fact was pointed out to the study team, changes were made in the clinic to improve confidentiality during interviews”. (Co Study Manager).*

### Enabling factors

In response to the question on what worked well in the functioning of the CAB, responses included the involving the community in the selection of the CAB members, advertising for membership as opposed to pre-selection, the use of former research participants as CAB members (two), involvement of the community stakeholders at an early stage during the study, holding of regular meetings, the use of existing structures to form the CAB, and the provision of training that increased the community understanding of research.

*“Using former research participants as CAB members is a good thing as these members are able to give their own experience with research”. (CAB member)”*

Choosing CAB members from within the community was viewed as positive especially from existing structures such as the Neighbourhood Health Committees (NHC) as these individuals were seen as already committed to contributing to the good of the community.

*“Because we are known by the community and people already trust us and know that we work for their benefit, they are ready to listen to us. It is important to choose influential people to serve on the CAB”. (CAB member)*

### Restricting factors

Using existing bodies and their corresponding members was associated with poorer commitment to the CAB due to other demands on these individuals and bodies. An additional factor was the fact that some existing CAB members had lower literacy levels and were unable to fully comprehend the nature of the study or concepts of research.

*“Our experience was that the use of professionals such as police officers or teachers did not work well as they were not always available to attend meetings”. (Senior Research Nurse)*

*“Though the NHC worked well and were committed it was sometimes difficult to work with those members whose reading and writing skills were limited. Even where the study was explained in their local language they were still not able to fully grasp the concepts and when providing information to the community they distorted the facts”. (Social Scientist).*

There were mixed responses about the use of community drama groups to sensitize communities about research. One research team member felt this was not effective as drama performances were attended mainly by children, whilst another research team member felt that this was an effective way of providing information. Additionally, lack of a designated focal person for the CAB at the health centre was seen as a negative experience as this lead to varied levels of support from the clinic staff for the CAB.

*“Because there was not one staff member that we worked with we sometimes did not get much support from the staff if they did not understand the work that we were doing”. (CAB member)*

Though no study provided stipends to the CAB members, transport reimbursement was provided for attending CAB meetings. Two of the research team members and two CAB members expressed the view that participation in the CAB should be based on a sense of voluntariness as opposed to relying on incentives. However two participants (research team and CAB member) indicated that a reliance on a spirit of voluntariness affected participation due to other commitments.

If the study team lacked commitment to the community, this undermined the ability of the CAB to function. Examples were given by two CAB members of study teams failing to keep to promises, address concerns of the CAB members or provide support for activities. Lack of insurance for the CAB members was cited by two respondents (CAB members) as being another negative factor.

*“Some form of insurance should be provided for CAB members to protect them in case of problems as a result of their work. One CAB member had his house burnt down after community members reacted to rumours that the study was related to Satanism”. (CAB member)*

One of the main functions of the CAB was perceived to be the link between the research team and the community, but two respondents (research team and CAB member) indicated that feedback from the community and the research team (respectively) was inadequate.

### Added value of CAB

Overall the majority of the responses focused on the positive value of including a CAB in the study through the use of descriptive phrases for the role as being a ‘bridge’, ‘belt’, ‘link’ or ‘intermediary’ between the research team and the community, “the eyes of the community and the ears of the study team”, and ‘a conduit of information’. The role of the CAB in reviewing the study protocol, informed consent documents and other study instruments was seen as crucial to ensuring that the study and methods were culturally acceptable and to ensure community acceptability of the study. However it was noted that for all the studies reviewed the CAB was engaged after the protocol was already developed and their role was rather to review of the documents with little genuine opportunity to contribute to the design of the study.

*“Even if we review the documents and give our suggestions these are not always taken on by the study team as they will say it is too late to change the study”. (CAB member)*

Both research team members and CAB members identified the valuable contribution of the CAB to developing good relationships between the community and the research team due to the trust that the community had in the members of the CAB when these were identified from within the community. Obtaining community input for the protocol and study instruments was seen as contributing to the overall sense of acceptance of the study by the community as well as increasing the level of knowledge and understanding of research in the community.

A comparison of the intra-team responses for the studies that had both a research team and CAB member interviewed (Table 5) showed that there was on the whole general agreement within the team. However, the response from the research team member differed in some cases from that of the CAB member in terms of identifying what worked well and did not work well. The CAB member tended to focus on aspects that impacted the way the CAB functioned while the research team member responses were broader involving both the role of the CAB as well as the aspects that affected the work of the CAB.

**Table 5:** Comparison of responses between the teams

Team	Participant	Worked Well	Did not work well	Added value of CAB	Recommendation
Team 1	Research Team	Involvement of stakeholders in community at early stage	Use of drama to sensitize community as mainly children attending	Involvement in protocol development leads to culturally acceptable questions, clarify rumours	Involve CAB in development of research question/idea instead of after approval of protocol
	CAB member	Involvement of community, transport reimbursement, travel	lack of internet access at clinic		lack of insurance for CAB members
Team 2	Research Team	Close link with clinic helped their role in dispelling rumours,	Feedback from community to research team not sufficient.	Give advice in how to disseminate information and results, Improve retention	Improve feedback from community to research team, better represent community
	CAB member	Close link with clinic and good relationship with study team, dispel rumours, myths, misconception	Need greater spirit of volunteerism and not dependent on incentives		More training on research,
Team 3	Research Team	Providing information to community to deal with rumours. Use of community drama groups	Lack of space for CAB meetings. Reliance on voluntarianism	Useful in tracking participants	
	CAB member	Support from staff, training, selection of members through adverts. Diverse group of participants	Use of professionals in CAB led to lack of commitment, involvement of Staff members in CAB		Provide insurance for CAB members. Involve participants in the study as CAB members.
Team 4	Research Team	Sensitization of community. Selection of CAB members through open method using adverts	Dependence on voluntarianism reduced commitment of CAB members	Helps to enter community, dispel rumours	Use research participants as part of CAB, involve them in sensitization of community
	CAB member	Involvement of CAB members from same community. Transport reimbursements. Dispelling rumours	Transport reimbursements inadequate		Involve CAB from conception of study and not only after protocol approved. Involve previous study participants
Team 5	Research Team	Dialogue with community through existing structures	Use of existing structures as CAB members as no control over quality of members	Role in improving retention in study, advocacy for participants (room, food	Improve role as representatives of the community. Ensure broad representation of community
	CAB member	Enhanced communication bet study team and community, helped to dispel rumours and reduce stigma	Feedback from research team inadequate, did not always fulfil promises		Need for capacity building of CAB members, should be involved in dissemination of results
Team 6	Research Team	Using existing structures as CAB members	Self-selection by existing structures as some members not literate and had difficulty understanding concepts of study	Improved recruitment and retention in study, sensitization of community	Use of existing structures as CAB members instead of a new structure to reduce conflicts
	CAB member	Involving existing structures to enter the community	Lack of a designated focal person Centre. Inadequate support to CAB		Ensure adequate support from Research Team

## Discussion

In the absence of a specific legal requirement to set up Community Advisory Boards when conducting clinical trials in Zambia, as has been reported for South Africa [5], these structures have been incorporated in the research either as a requirement by the sponsor or based on the practice of the research team. Indeed, in the initial review of the approved studies at least two studies that did not have a CAB would have had one were this a requirement for approval of a clinical trial with an intervention arm.

All the CABs reviewed had been set up using a broad community model whereby membership was either chosen from the general community through advertisement of the CAB or through nomination and election from existing community structures.

A noted benefit of using community members from the community where the research is taking place was the level of trust and acceptance of the members of the committee by the wider community. As in the experience in Thailand, Zimbabwe, [6] and South Africa [5] an added advantage was their inside knowledge and understanding of the community and their influence on the community.

However when existing community structures are used to form the CAB, selection of the members should take in to account commitment of the members to community work and balance this with basic skills such as comprehension and understanding given the specialised nature of research. For example, a research study in Bagamoyo, Tanzania included literacy as a requirement for membership in the CAB [7]. And elsewhere [6], a gap in understanding between the researcher and the CAB member was noted to be a retention barrier for continuity of membership.

As reported for other CABs [2, 6, 13], a major function reported in this study was that of facilitating communication between the research team and the community. The review of protocols, questionnaires, informed consent forms, and other study related documents was also perceived to be an important function of the CAB as ten of the fourteen respondents cited this as a role for the CAB. Involving the CAB in the review of these documents was noted to contribute to community ownership of the study and ensured cultural sensitivity of the study. However the experience of all groups interviewed was that this was done after the protocol was developed in some cases or focused on how to conduct the study as opposed to involving them in the conceptual phase of the study. Involving the community in the earlier phases of protocol development where feasible will help to ensure incorporation of the community based participatory approach to research that includes true community participation and ensures that the community’s concerns and priorities are included in the design of the research [8]. Involving the CAB at an earlier stage of protocol development is one approach to ensure that the CAB functions as true partners in the research endeavour as opposed to being advisors. Providing advice does not guarantee that the advice will be accepted and utilized (8). Involving the CAB in the more substantive aspects of the research endeavour will allow them to have a broader role than functioning as a link between the research team and the community for the sharing of information. This will in turn, allow the CAB be more effective in reducing exploitation of communities [13]. On the other hand, this may make the CAB seem more a part of the study team thereby compromising their independence. Thorough training and fully comprehending research may help to uphold CAB’s necessary independence.

An added value of the CAB was their ability to contribute to recruitment and retention of study subjects as has been reported for a CAB for tuberculosis control in KwaZulu-Natal [11].

Although none of the groups included in this study provided stipends as was reported for a US-based CAB [2], monetary support was provided in the form of transport reimbursements for attendance at meetings as has been reported elsewhere [6]. Other support provided included capacity building in research, refreshments, attendance at Conferences, among others. The perception of this practice in this study was mixed as some respondents felt that support was important in order to ensure commitment on the part of the CAB member whilst others felt this would compromise the role of the CAB member due to dependence on the study team for this benefit. The use of transport reimbursements therefore should be handled judiciously to avoid the appearance of the CAB leaning more towards the study team than the community. Providing transport reimbursements for meetings conducted at the clinic also has the potential to distort the operation of other groups associated with the clinic such as the NHCs and Community Treatment supporters who usually do not get reimbursed when attending meetings at the clinic. Further research is needed on the impact of transport reimbursements and other incentives for the CAB in terms of their ability to function as an independent body.

While it has been recognised that compensation of CAB members for their participation is associated with greater commitment to the CAB [2], when this is provided by the research group there is a possibility that the CAB members may be influenced in their decision making in favour of the research team. The challenge then is to balance the need for compensation with the requirement for maintaining the independence of the CAB. One approach is to consider the CAB members as volunteers whose motivation for participation arises from altruism [11, 14]. However the reality of situation in many communities is that the individuals available to participate in a CAB may be those with are unemployed and therefore have challenges meeting their daily needs [11, 15]. One potential solution is to set up CABs that are not linked to a specific study [6, 7, 16, 10] such that the CAB will not be dependent on the study team to provide whatever type of support is provided, whether it be a stipend or reimbursement for transport cost associated with attendance at CAB meetings. One advantage of using a non-study specific CAB is that this allows for a relationship to be established between the CAB and the community that will last beyond the duration of a single study, leading to a greater sense of trust between the two [17].

### Limitations of the study

One of the limitations of the study is that it is possible that other studies approved during the period under review were not identified in the initial search for potential studies with a CAB for the period 2008–2012. In addition the study did not include interviews of community members to obtain their perspective on the functioning of the CAB and only focused on the perspectives of the study team and CAB members. As some of the studies were completed by the time the interviews were done there is the possibility that the responses to the questions were affected by recall bias.

## Conclusion

The CABs reviewed in this study were all established using the broad community model. The experience with the use of CABs in Lusaka, Zambia was constructive and affirmative, with the CAB being viewed as an important link between the research team and the community, ensuring that the research was acceptable and that community engagement occurred with the research process. Community input into the terms of reference was achieved by either the involvement of existing community structures or existing CABs in conjunction with the research team. Membership of the CAB was selected from the residents of the community where the research was taking place and this ensured that the community had trust and confidence in the CAB.

The CAB was recognized to be important in sensitization of the community to the particular research study as well as serving as the link to improve communication between the research team and community. An additional role that was identified was in reviewing the protocol and study instruments to ensure that the study was acceptable to the community. As a result of the trust the community had in the CAB members they were able to improve the recruitment and retention in the study.

In order to effectively represent the interests of both the community and the study team the CAB should function independently of the research team. As the CABs were appointed by the research teams and support for their activities was provided by the research team, including transport reimbursements, there is the possibility that the CAB may not be totally independent of the research team. On the other hand a total reliance on voluntary participation was noted to have the potential to have a negative impact on the commitment of the CAB members. The concept of community wide representatives on a research advisory board as opposed to study group specific boards is a concept that should be explored as this would ensure greater independence of the CAB from the research team.

## Abbreviations

CAB: Community Advisory Board
CTU: Clinical Trials Unit
NHRS: National Health Research system
NHC: Neighbourhood Health Committee
UNZABREC: University of Zambia Bioethics Research Committee
